# A Novel Transgenic Mouse Line for Tracing MicroRNA-155-5p Activity *In Vivo*

**DOI:** 10.1371/journal.pone.0128198

**Published:** 2015-06-01

**Authors:** Krung Phiwpan, Jie Guo, Wei Zhang, Tanyu Hu, Bhargavi M. Boruah, Jianhua Zhang, Xuyu Zhou

**Affiliations:** 1 CAS Key Laboratory for Pathogenic Microbiology (CASPMI), Institute of Microbiology, Chinese Academy of Sciences, Beijing, China; 2 University of Chinese Academy of Sciences, Beijing, China; 3 National Laboratory of Biomacromolecules, Institute of Biophysics, Chinese Academy of Sciences, Beijing, China; South Texas Veterans Health Care System and University Health Science Center San Antonio, UNITED STATES

## Abstract

MicroRNA-155 (miR-155) plays significant role in various physiological processes involving both innate and adaptive immunity. miR-155 expression level changes dynamically during various immune responses. However, current approaches for miR-155 detection at the RNA level do not precisely reflect the real-time activity. Herein, we generated a transgenic mouse line (R26-DTR-155T) for determination of miR-155-5p activity *in vivo* by inserting miR-155-5p target sequence downstream of a reporter transgene comprising Diphtheria Toxin Receptor and TagBlue fluorescence protein. Using this approach, R26-DTR-155T mice were able to measure variation in levels of miR-155-5p activity in specific cell types of interest. The DTR expression levels were inversely correlated with the endogenous miR-155 expression pattern as detected by quantitative RT-PCR. Our data demonstrate a novel transgenic mouse line which could be useful for tracing miR-155-5p activity in specific cell types through measurement of miR-155-5p activity at single cell level.

## Introduction

MicroRNA-155 (miR-155) is processed from the non-protein coding transcript of the *BIC* (B cell Integration Cluster) gene located on chromosome 21 in human and chromosome 16 in mice [1, 2]. miR-155, like other microRNAs (miRNAs), is transcribed by RNA polymerase II to generate primary transcripts (pri-miR-155) that is processed in the nucleus to generate miRNA precursors (pre-miR-155). The pre-miR-155 is then exported into the cytoplasm and is further processed by *Dicer* leading to 23 nucleotides long duplex miRNA [3]. Based on the stability of the 5’ end, one strand (passenger miR-155) of the miRNA duplex is released and degraded while the other strand (guide strand or mature miR-155), is retained and loaded into the RNA-induced silencing complex (RISC) which binds to target mRNAs as well as regulates gene expression by either repressing protein translation or inducing mRNA degradation. Both arms of pre-miR-155 can develop into mature miR-155-5p or miR-155-3p based on the selection of either 5’ or 3’ strand respectively [4]. However, the expression level of miR-155-5p is reported to be ~20–200 fold higher than that of miR-155-3p [5].

Although miR-155 was initially described as an oncogenic miRNA [6], the generation of knockout mice lacking BIC/miR-155 highlights the critical role of miR-155 in both innate and adaptive immunity [7]. Moreover, miR-155-deficient dendritic cells have been reported to lose efficiency during antigen presentation [8]. In addition, miR-155 regulates IFN-γ production in natural killer cells [9], controls differentiation of CD4 T helper cell subsets into Th1, Th2, and Th17, as well as promotes development of Treg cells [10, 11]. In CD8 cells, miR-155 is important for the development of T effector function and the memory cytotoxic lymphocyte (CTL) formation. Moreover, miR-155 is essential for normal production of isotype-switched, high-affinity antibodies in B cells [12–14].

Expression of miR-155 changes dynamically during immune responses and its overexpression is linked to various diseases ranging from hematological malignancies, cancer, viral infections and autoimmune diseases [15]. Current approaches for miR-155 detection are mainly based on quantitative reverse transcription PCR (qRT-PCR), microarray, and deep sequencing [16, 17]. miR-155 KO mice, in which the exon2 of bic/miR-155 gene was replaced by lacZ reporter gene, also allow to study the pri-miR-155 expression *in vivo* [14]. These methods represent miR-155 expression at RNA level and do not reflect the real-time function of miR-155 activity in living cells. Recently, Schug and colleagues [18] have reported that assessment of expression at RNA level alone does not reflect miRNA activity and is likely to be influenced by multiple factors including contributions of RNA binding proteins, ratio of mRNA to target miRNAs, flanking sequence homology, and change in subcellular localization of miRNA, suggesting unique regulation of miRNA function *in vivo* [18, 19]. Attempts have been made to study miRNA activity in living systems, through development of miRNA sensors based on OFF-system by inserting miRNA target sequence into the 3’-UTR of reporter genes such as lacZ [20], GFP [21] and luciferase [22, 23]. The endogenous miR will bind to the reporter transcript and downregulate its expression. Another miRNA sensor is based on ON-system by inserting miR binding sites into the 3’-UTR of repressor genes together with a reporter cassette under the regulation of the repressor. The reporter is switched-on by the endogenous miR of interest which degrades the repressor mRNA [24, 25]. Although these sensors can check miRNA activity *in vivo*, their usefulness has not been fully evaluated in living single cells.

In this study, we generated novel miR-155-5p sensor transgenic mice, namely R26-DTR-155T, based on a combination of BAC transgenic approach and a Cre-Lox system by inserting miR-155-5p target sequence into 3’-UTR of DTR-BFP reporter gene. The miR-155-5p/DTR-BFP target-reporter gene module was further placed downstream of the LoxP-STOP-LoxP cassette driven by Rosa26 (R26) promoter. Using this approach, the level of miR-155-5p activity could be effectively determined in these transgenic mice. Furthermore, R26-DTR-155T mice also provided tissue-specific sensing of miR-155 activity by selection of a tissue-specific promoter driven Cre expression. In addition, it could offer the possibility to manipulate a particular cell population according to the expression pattern of miR-155-5p in the distinctive-expressing cells. Our results indicated that R26-DTR-155T transgenic mice may serve as useful tools to uncover miR-155-5p activity and its function in various cell-subsets *in vivo*.

## Materials and Methods

### Plasmid constructs

For construction of miR-155-5p sensor vector (pDTR.BFP-155T-N1) and recombinant Bacterial Artificial Chromosomes (BACs), we synthesized monkey Diphtheria Toxin Receptor (DTR) and TagBlue Fluorescence Protein (BFP) in pUC57 cloning vector (GenScript, Piscataway, NJ) and used them as template to amplify the DTR or BFP gene sequence. Both amplifications were performed through overlapping PCR to create DTR-BFP fusion reporter gene. The fusion reporter gene was cloned into pEGFP-N1 vector (Takara Clontech) in place of EGFP sequence, henceforth, termed as pDTR.BFP-N1 vector. miR-155-5p target gene (Accession no. MIMAT0000165) was designed and cloned into pUC57 vector (GenScript, Piscataway, NJ) containing four copies of the sequence (underlined) linked with four nucleotides in between as follows: ACCCCTATCACAATTAGCATTAACGATACCCCTATCACAATTAGCATTAACGATACCCCTATCACAATTAGCATTAACTACACCCCTATCACAATTAGCATTAA. The 4×miR-155-5p target (118 bp in length) fragment was subsequently digested with restriction enzymes *EcoRI* and *NotI* and cloned into the pDTR.BFP-N1 vector within similar restriction sites to generate pDTR.BFP-155T-N1 vector. Both pDTR.BFP-N1 and pDTR.BFP-155T-N1 vectors were then digested with *NotI* and *XhoI* and ligated downstream of LoxP-STOP-LoxP cassette into pBigT vector (Addgene plasmid 21270) [26], respectively. In order to perform homologous recombination with BAC (RP24-85L15) (CHORI, Oakland, CA, USA), the recombinant pBigT vector was used as template to amplify the cassette of a 4.7 kb transgene (LoxP-STOP-LoxP-DTR.BFP-155T or LoxP-STOP-LoxP-DTR.BFP) comprising of R26 arm sequence, having 200 bp upstream and 400 bp downstream. The transgene in recombinant BACs was checked by *XhoI* restriction enzyme as well as DNA sequencing before microinjection.

To construct the miR-155 expression vector (pEF-BOS-EX-miR-155), mouse miR-155 gene was amplified using the following primers: F 5’-CGGGATCCTGAACCGTGGCTGTGTTAAA-3’ and R 5’-GCTCTAGAAGAATGGCCGTCCTGAATTT-3’. The amplified products were digested with *BamHI* and *XbaI* and then cloned into the pEF-BOS-EX vector [27] within the respective restriction enzyme sites.

To generate pmirGLO Dual-Luciferase miR-155-5p target expression vector (pmirGLO-4×miR-155-5pT), 4xmiR-155-5pT-pUC57 cloning vector was digested with *EcoRI* and *HindIII* to transfer 4×miR-155-5p target sequence into the *EcoRI* and *HindIII* site of pBluescript II SK+ phagemide (Stratagene, La Jolla, CA). The target sequence was subsequently digested with *SacI* and *XhoI* to clone into the pmirGLO vector (Promega).

### Generation of R26-DTR-155T transgenic mice

The final BAC constructs were microinjected into pronuclei of fertilized B6×DBAF1 mouse oocytes to generate R26-DTR-BFP-miR-155-5p target mice (R26-DTR-155T mice) or R26-DTR-BFP mice (R26-DTR mice). The R26-DTR-155T mice were crossed with CMV-Cre mice (Tg(CMV-Cre)1Cgn) to obtain CMV-Cre x R26-DTR-155T mice for screening the DTR reporter protein and were used for determining miR-155-5p activity in distinct cell lineages. To determine miR-155-5p activity in Treg cell subsets, the R26-DTR-155T mice were crossed with Foxp3-GFP-Cre x R26-YFP mice to achieve Foxp3-GFP-Cre x R26-YFP x R26-DTR-155T mice. The Foxp3-Cre mice are Tg(Foxp3-EGFP/cre)1cjbs strain and were crossed with R26-YFP knockin mice as described previously [28]. All mice were housed and bred under specific pathogen-free conditions at the Beijing Laboratory Animal Research Center in accordance with the guidelines for care and use of laboratory animals established by the Beijing Association for Laboratory Animal Science. All animal procedures were conducted in accordance with the “Regulation of the Institute of Microbiology, Chinese Academy of Sciences of Research Ethics Committee”. The protocol was approved by the Research Ethics Committee of Institute of Microbiology, Chinese Academy of Sciences (PZIMCAS2012002).

### Validation of miR-155-5p-OFF system *in vitro*

miR-155-5p target reporter assay by luciferase assay was performed in 24-well plates. pmirGLO-4×miR-155-5pT and pEF.BOS.EX-miR-155, pEF.BOS.EX-miR-146a or pEF.BOS.EX-empty vector were mixed at a molar ratio of 1:1 in Opti-MEM (Promega) followed by mixing with Lipofectamine2000 (Invitrogen). After 20 minutes incubation at room temperature, the mixture was gently added into HEK293T cells (1×10^5^ cells/well). The transfected HEK293T cells were cultured in DMEM (Dulbecco’s modified Eagle’s medium, Gibco, Life Technologies) supplemented with 10% fetal bovine serum, GlutaMax, 100U/mL penicillin, and 100 μg/mL streptomycin at 37°C in 5% CO_2_ for 24 hours, then cells were analyzed for luciferase activity using Dual-Glo Luciferase Assay System (Promega) according to the manufacture’s protocol.

For the miR-155-5p target reporter assay by flow cytometry, HEK293T cells were placed at 1×10^5^ cells/well in 24-well plates and co-transfected with pDTR.BFP.155T-N1 and pEF.BOS.EF-miR155 or pEF.BOS.EX-empty vector at various molar ratios of 1:5, 1:1 and 5:1 using Lipofectamine2000 (Invitrogen). After 48 hours post transfection, cells were analyzed using flow cytometry. Data were collected and processed using FACS analysis software (FlowJo).

For evaluating sensitivity of the R26-DTR-155T system, HEK293T cells were placed at 1×10^5^ cells/well in 24-well plates and co-transfected with 400 ng/well of pDTR.BFP.155T-N1 along with increasing amounts of miR-155-5p mimic or miR control mimic (RiboBio, China) in the presence of Lipofectamine 2000 (Invitrogen). 48 hours post transfection, cells were analyzed using flow cytometry. Data were collected and processed using FACS analysis software (FlowJo).

### Quantitative real-time PCR

Total RNA from each sorted cell type of interest from Foxp3-GFP-Cre x R26-YFP mice was extracted by Trizol reagent (Invitrogen). The Bulge-Loop miRNA qPCR Primer Set (RiboBio, China) was then used to detect miR-155 expression by qRT-PCR using QuantiTect SYBR Green PCR +UNG Kit (QIAGEN,204163). The overall reaction, in a total volume of 10 μL, consisted of 5 μL Master Mix, 200 nM of each primer, and 2 μL of the diluted cDNA product. After 2 min at 50°C, the DNA polymerase was activated at 95°C for 15 min, followed by 45 cycles at 95°C for 15 s, 60°C for 30 s, and 72°C for 30 s. U6 primer was used to normalize target miR-155 expression. Relative expression levels for miR-155 were determined using the 2^-ΔΔCt^ method. All reactions were performed three times using a LightCycler480 System (Roche Applied Science).

### Antagonism of miR-155 function

Antagomir-155 was purchased from RIBOBIO (Guangzhou, China). Lymph node cells of CMV-Cre x R26-YFP x R26-DTR-155T mouse founder 47 were stimulated with 0.5 μg/mL anti-CD3 (2C11) in RPMI-1640 (Gibco, Life Technologies) supplemented with 10% fetal bovine serum, non-essential amino acids (NEAA), 2-ME, 100U/mL penicillin, and 100 μg/mL streptomycin at 37°C in 5% CO_2_ for 48 hours. The activated cells were then washed and seeded at 1×10^6^ cells/well in 24-well plates to perform transfection with 200 nM antagomir-155 or 200 nM antagomir-142 (negative control) using Lipofectamine2000 (Invitrogen) according to the manufacture’s protocol. After 18 hours of transfection, cells were analyzed by FACS Calibur (BD Biosciences).

### DTR staining for determining miR-155-5p activity

For cell surface staining, lymph node cells at a density of 2×10^6^ were first stained with 4 μg/mL anti-DTR-biotin (R&D cat.BAF259) and then stained with Streptavidin-PE (BioLegend cat.405204) along with either of the following fluorochrome-conjugated antibodies: anti-CD4-PerCP clone GK1.5 (Tianjin Sungene Biotech Co.), anti-CD44-APC clone IM7 (eBioscience), anti-B220-PerCP-cy5.5 clone RA3-6B2 (eBioscience), anti-CD62L-APC clone MEL-14 (BioLegend), and anti-CD8-Brilliant Violet 605 clone 53–6.7 (BioLegend). For intracellular Foxp3 staining, cells were first treated with the antibodies specific for cell surface and later stained with anti-Foxp3-Alexa Fluor488 clone FJK-16s (eBioscience) using the intracellular-staining kit from eBioscience. Cells were analyzed on Fortessa (BD Biosciences) or FACS Calibur (BD Biosciences). Data were analyzed using FACS analysis software (FlowJo).

### Diphtheria toxin (DT) treatment

All mice were aged 8–16 weeks at the onset of experiment. Each mouse was i.p. injected with 50 μg/kg body weight of DT from *Corynebacterium diphtheria* (Calbiochem, San Diego, CA, USA) on two consecutive days, day0 and day1. Blood samples were taken from each mouse prior to injection, on the indicated days. The peripheral blood was lysed, stained with anti-CD4 and examined through FACS Calibur.

## Results

### *In vitro* validation studies

**Fig 1:**
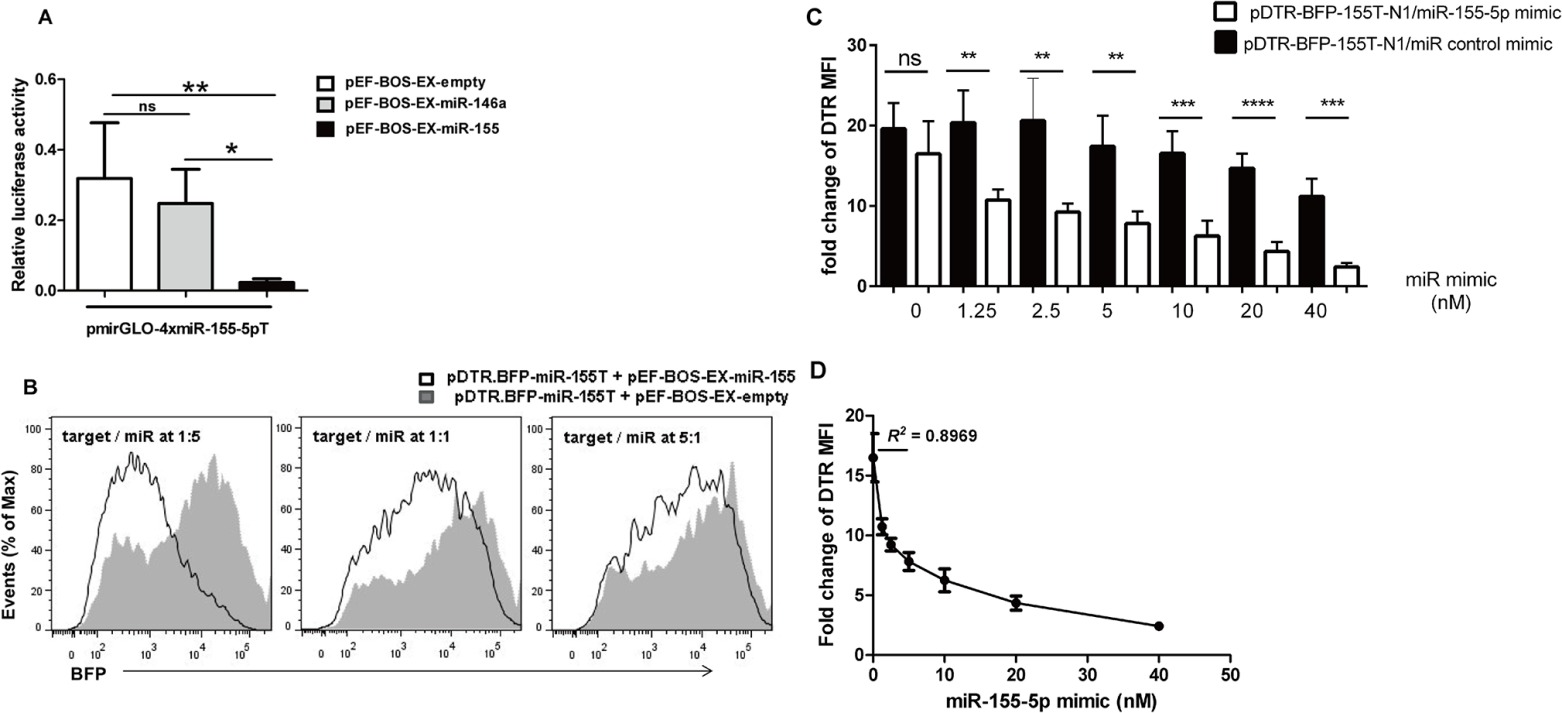
Validation of miR-155-5p-OFF system *in vitro*. (A) Validation of miR-155-5p target using luciferase assay, HEK293T cells were transfected with pmirGLO-4xmiR-155-5pT plus pEF-BOS-EX-miR-155, pEF-BOS-EX-miR-146a, and pEF-BOS-EX-empty vector (control), respectively, at a molar ratio of 1:1. (B) Validation of miR-155-5p target by flow cytometry, expression vector for miR-155 (pEF-BOS-EX-miR-155) or a control vector (pEF-BOS-EX) was co-transfected into HEK293T cells with an expression vector for miR-155-5p target reporter sequence (pDTR.BFP-155T) at various molar ratios. The expression of BFP reporter signal was analyzed through flow cytometry after 48 hours of transfection. (C and D) Sensitivity of miR-155-5p-OFF system. Fold change of mean fluorescent intensity of DTR expression in HEK293T cells transfected with pDTR.BFP-155T-N1 plasmid with increasing concentrations (0, 1.25, 2.5, 5, 10, 20, and 40 nM) of synthetic miR-155 or miR control. Significance was calculated by Student’s *t*-test using GraphPad Prism 5 software. N = 3, ns: not significant; *P<0.05; **P<0.01, ***P<0.001, ****P<0.0001.

MicroRNA activity in gene regulation mainly occurs through the interaction of miRNA with the 3’-UTR of its target gene. To assess the specificity of the miR-155-5p-OFF system, miR-155-5p target was evaluated by inserting four tandem copies of a 23-bp sequence with perfect complementarity to miR-155-5p into the 3’-UTR of the firefly luciferase gene. This strategy of using multiple copies of miRNA target sequence has been well validated [21]. The miR-155-5p target was initially validated by a conventional luciferase reporter assay in transfected HEK293T cells with a pmirGLO carrying miR-155-5p target and vector expressing miR-155 or miR-146a (as a miRNA control). The results showed that the miR-155-5p target was responsible for overall miR-155-5p interaction leading to decreased luciferase signals compared to HEK293T cells transfected with miR-146a (Fig 1A). Next, we constructed the miR-155-5p sensor by inserting the 4×miR-155-5p target sequence into the 3’-UTR of a DTR.BFP fusion reporter gene (termed as DTR.BFP-155T). FACS analysis showed that miR-155-5p was able to knock down the BFP reporter signal leading to decrease in expression level of BFP in dose dependent assays (Fig 1B). To further determine sensitivity of the miR-155-5p-OFF system in response to expression of miR-155-5p, HEK293T cells were transfected with pDTR.BFP-155T-N1 along with varying amounts of synthetic precursor miR-155 ranging from 0 to 40 nM (Fig 1C). It showed that the fold change of DTR expression from HEK293T cells transfected with pDTR.BFP-155-N1 was inversely correlated with increasing amounts of synthetic miR-155. The linear relationship between miR-155-5p level and DTR expression was found to be correlated (R^2^ = 0.8969), with varying concentration of miRNA ranging from 0 to 2.5 nM (Fig 1D).

### Generation and Characterization of miR-155-5p sensor transgenic mice

**Fig 2:**
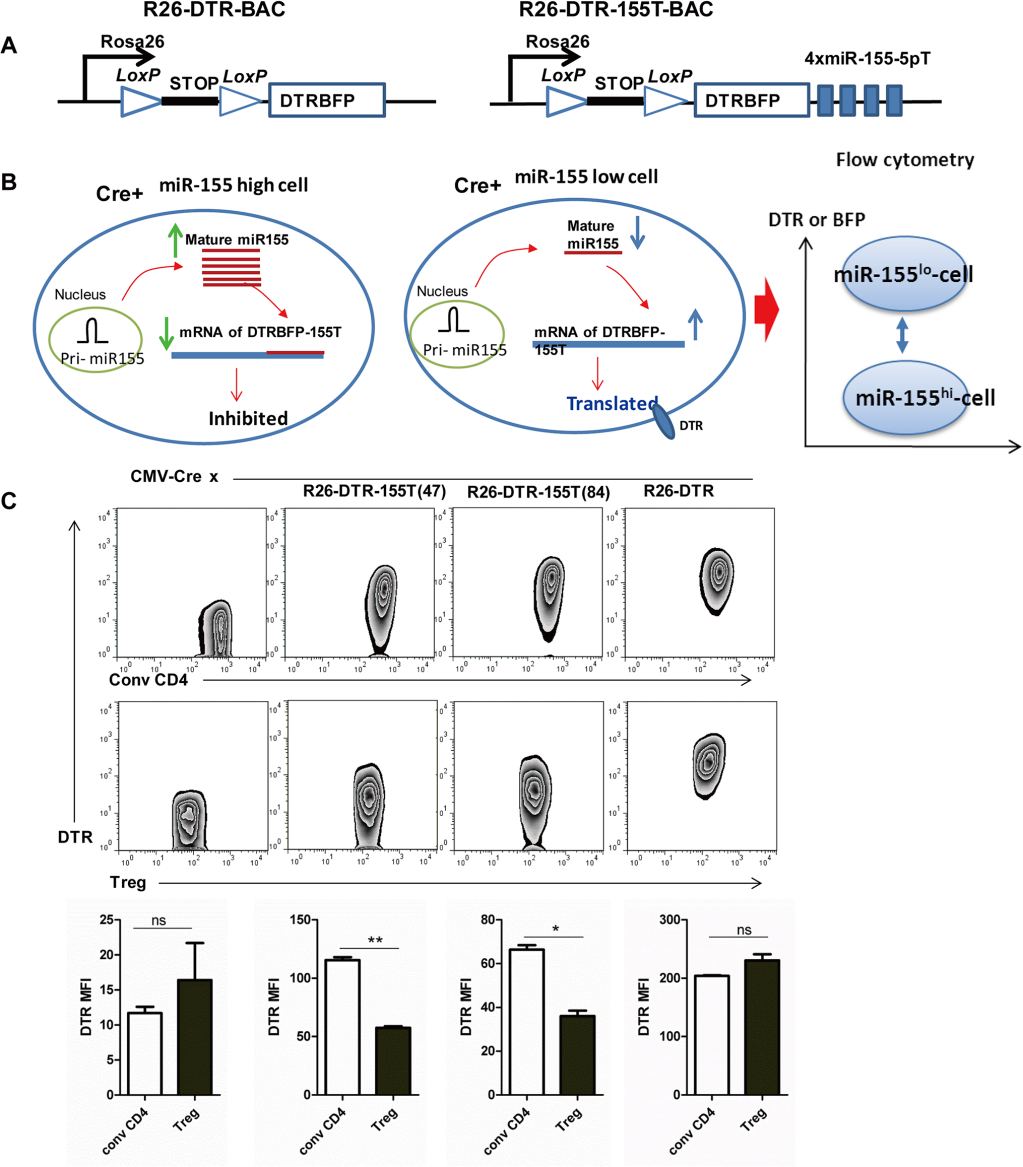
Generation and Characterization of miR-155-5p sensor transgenic mice. (A) Schematic illustration of the BAC transgenic constructs. Left panel-R26-DTR-BAC lacking miR-155-5p target sequence. Right panel-R26-DTR-155T-BAC containing 4×miR-155-5p target sequence in the 3’-UTR of DTR-BFP fusion gene. The fusion cassette gene was then placed downstream of a STOP element flanked by LoxP sites driven by Rosa26 promoter. (B) Schematic illustration of the determination of miR-155-5p activity in single living cell by flow cytometry. The relative activity of miR-155-5p will be inversely proportional to the expression level of DTR or BFP. (C) Phenotype of R26-DTR-155T mice. Figures in the upper panel show FACS profiling of DTR expression in conventional (conv) CD4-gated cells and Treg-gated cells of different mice as shown. Two founders, 47 and 84, of the R26-DTR-155T mice and a R26-DTR mouse were crossed with CMV-Cre mice; lymph node (LN) cells of the littermates were stained with anti-DTR-Biotin and then conjugated with Streptavidin-PE along with anti-CD4 and anti-Foxp3 (intracellular staining) and analyzed through FACS Calibur. Figures in the lower panel represent DTR MFI of indicated cells (shown in the figures of upper panel). Significance was calculated by Student’s *t*-test using GraphPad Prism 5 software. N = 3, ns: not significant; *P<0.05; **P<0.01.

With an aim to generate miR-155-5p sensor transgenic mouse providing tissue specific determination and ablation, the 1463 bp long DTR.BFP-155T fragment was inserted downstream of the LoxP-STOP-LoxP element in order to prevent transcription of the reporter gene. Further, the whole cassette, LoxP-STOP-LoxP-DTR.BFP-155T, was inserted downstream of a transcriptional start site at the ubiquitously expressed R26 locus (Fig 2A). The final modified BAC constructs were then microinjected into the pronuclei of fertilized oocytes of B6×DBA2F1 mice to generate R26-DTR-155T sensor transgenic mice. R26-DTR mouse carrying an identical transgene with the exception of miR-155-5p target sequence was used as control (Fig 2A). We obtained two founders, 47 and 84, for R26-DTR-155T mice and one for R26-DTR mouse.

To characterize the founder mice, three founders were first crossed with CMV-Cre mice that express Cre recombinase driven by the cytomegalovirus minimal (CMV) promoter. In the resultant mice, deletion of LoxP-flanked stop cassette occurred in all tissues including germ cells leading to the transcription of the reporter gene in every living single cell [29]. In miR-155 -5p high-expressing cell, miR-155-5p binds to target mRNAs and inhibits or degrades protein translation. However, in case of miR-155-5p low-expressing cell, the overall concentration of miR-155-5p has no significant effect upon the translation process. When examine with flow cytometry, the activity of miR-155-5p will be inversely proportional to the expression level of DTR or BFP reporter protein (Fig 2B). The BFP signal was detectable in all mice, whereas the DTR had a much stronger signal; therefore, we selected the DTR as a reporter in this study (data not shown). DTR expression in both conventional (conv) CD4 cells and Treg cells from two founders of miR-155-5p sensor transgenic mice was significantly lower compared to the control. Since founder 47 was much lower in expressing the DTR reporter than founder 84 (Fig 2C), it might indicate that different copy numbers or integration sites of the transgene into the chromosome resulted in unequal yield of miR-155-5p target reporter mRNA [30, 31]. Importantly, DTR expression by Treg was significantly decreased from R26-DTR-155T mice only and not from R26-DTR mice (control), in comparison with expression by conv CD4 cells.

### Inhibition of miR-155-5p rapidly releases DTR expression

**Fig 3:**
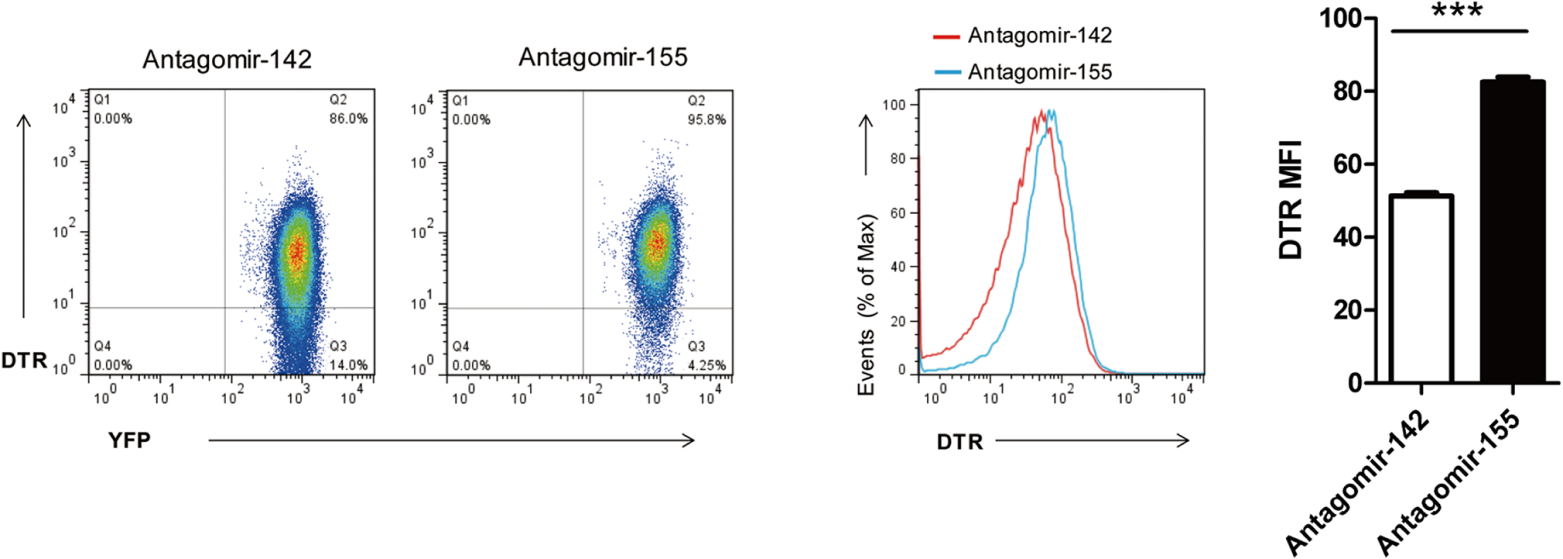
Inhibition of miR-155-5p with antagomir-155 rapidly releases DTR expression. LN cells of CMV-Cre x R26-YFP × R26-DTR-155T mice founder 47 were stimulated with anti-CD3 for 48 hours. The activated cells were then transfected with 200 nM antagomir-155 or 200 nM antagomir-142 as negative control. FACS analysis was performed after 18 hours of transduction for detecting DTR expression. Left panel shows the dot plots of DTR expressing cells transduced with indicated antagomir. Middle panel shows the overlaid histograms of DTR expression between antagomir-155 and antagomir-142 transfected cells. Right panel shows DTR MFI of indicated cells. Significance was calculated by Student’s *t*-test using GraphPad Prism 5 software. N = 3, ****P<0.0001.

To test whether the low DTR expressing cells was the effect of high level of miR-155-5p activity, we carried out experiments with the transduction of antagomiR-155 also known as anti-miR-155, which was used to silence endogenous miR-155-5p. Flow cytometry analysis showed that the expression of DTR was increased significantly once miR-155-5p was inhibited (Fig 3). These results indicated that the expression level of DTR was significantly influenced by the level of endogenous miR-155-5p activity.

### Detection of miR-155-5p activity by R26-DTR-155T mice in distinct cell lineages

**Fig 4:**
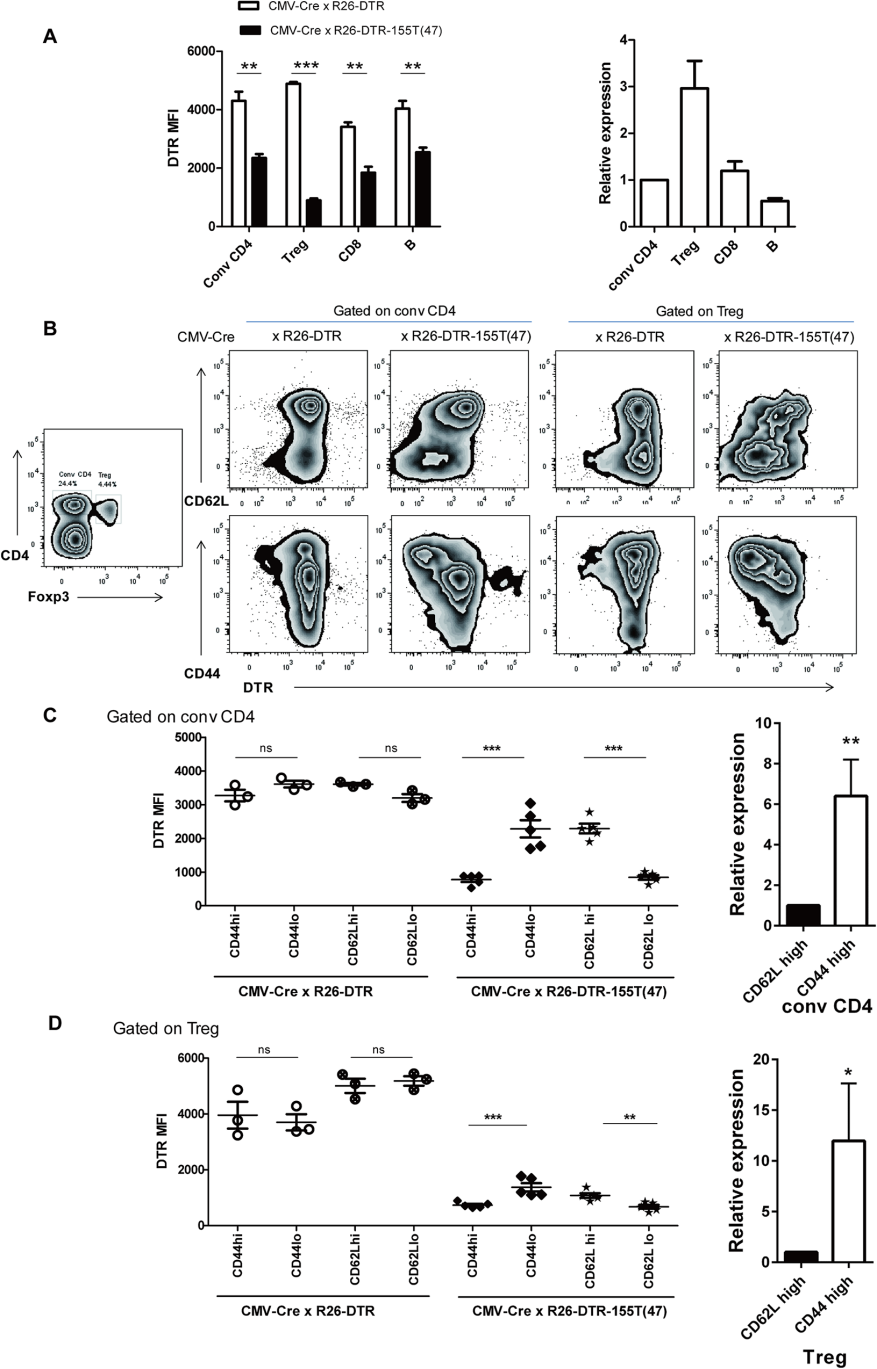
Relative difference of DTR expression in R26-DTR-155T mice reflects miR-155-5p activity in different cell lineages. (A) miR-155-5p activity in specific cell types of interest. LN cells from CMV-Cre x R26-DTR-155T mice or CMV-Cre x R26-DTR mice were stained with anti-DTR along with either anti-CD4, anti-Foxp3 (intracellular staining), anti-CD8 and anti-B220 for conventional CD4 (CD4+Foxp3-), Treg (CD4+Foxp3+), CD8 and B cells respectively. DTR expression was shown by mean fluorescence intensity (MFI). Data represent mean ±SD (N = 3 for CMV-Ce x R26-DTR mice and N = 5 for CMV-Cre x R26-DTR-155T mice). Figure on the right represents endogenous expression levels of miR-155 in indicated cells detected by qRT-PCR. (B) FACS profiling represents the pattern level of miR-155-5p activity of conv CD4-gated cells and of Treg-gated cells for CD44^high^ or CD44^low^ cells and CD62L^high^ or CD62L^low^ cells. (C-D) Comparison of DTR MFI of conv CD4-gated cells and Treg-gated cells in CD44^high^ or CD44^low^ cells and CD62L^high^ or CD62L^low^ cells from R26-DTR mice and R26-DTR-155T mice. Each symbol represents one mouse and small horizontal lines indicate the mean. Figure on the right represents endogenous expression levels of miR-155 in indicated cells (in naïve conv CD4 (CD4+ CD62L^high^ YFP-), effector conv CD4 (CD4+ CD44^high^ YFP-), in naïve Treg (CD4+ CD62L^high^ YFP+, effector Treg (CD4+ CD44^high^ YFP+)), effector Treg (CD4+ CD44^high^ YFP+)) of Foxp3-GFP-Cre x R26-YFP mice detected by qRT-PCR. Significance was calculated by student t-test using GraphPad Prism version 5 software. N = 3, ns; not significant, *P<0.05, **P<0.01, ***P<0.0001.

Next, we took advantage of CMV-Cre x R26-DTR-155T and CMV-Cre x R26-DTR control mice to determine the activity of miR-155-5p in conv CD4 (CD4+Foxp3-), Treg (CD4+Foxp3+), CD8, and B cells under homeostatic condition (Fig 4A). Only Treg cells showed strikingly decreased level of DTR expression in resting conditions, which is consistent with previous deep sequencing reports [32, 33] and further confirmed by qRT-PCR analysis (Fig 4A).

It has been reported that miR-155 is highly expressed in effector or memory Treg cells compared to lower expression levels in both naïve Treg cells and naïve conventional CD4 cells [33]. To determine miR-155-5p activity in effector cells, we used CD44 and CD62L as marker to define them. We observed that CD44^high^ cells or CD62L^low^ cells of CMV-Cre x R26-DTR-155T mice showed low level of DTR expression in both conv CD4 cells and Treg cells compared to that of CMV-Cre x R26-DTR mice (Fig 4B). The low level of DTR expression in CD44 ^high^ cells or CD62L ^low^ cells was significant different from CD44^low^ cells or CD62L^high^ cells in both conv CD4-gated cells and Treg-gated cells of CMV-Cre x R26-DTR-155T mice (Fig 4C and 4D). The expression of DTR in naïve cells and effector cell of conv CD4+ and Treg cells from CMV-Cre x R26-DTR-155T mice was inversely correlated with the expression level of miR-155 in those cells from Foxp3-GFP-Cre x R26-YFP mice detected by qRT-PCR. These results demonstrate the response by CMV-Cre x R26-DTR-155T mice to distinct levels of miR-155-5p activity, which implies that effector cells have higher level of miR-155-5p activity than naïve cells.

**Fig 5:**
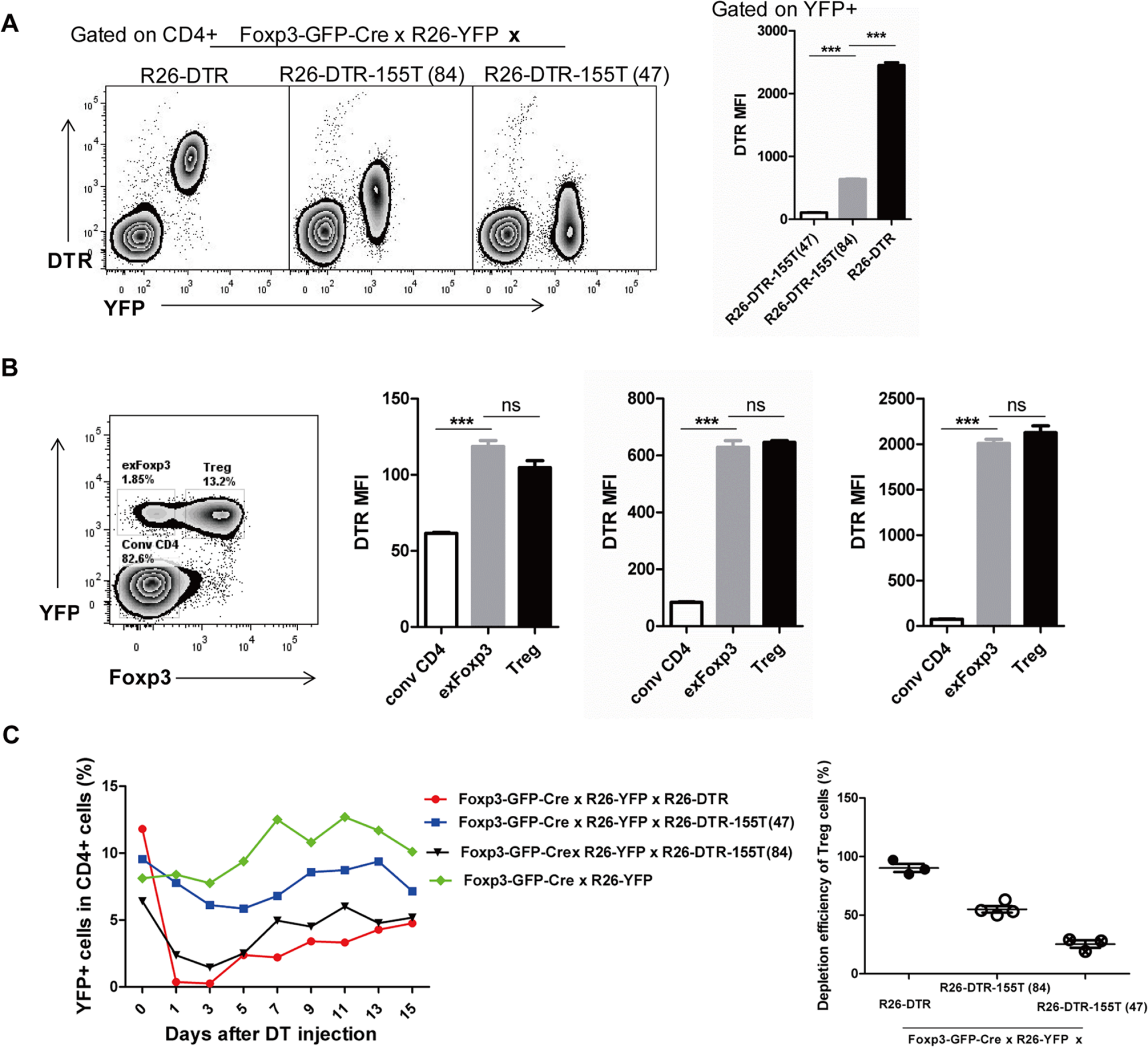
Activity of miR-155-5p in Treg cell subsets. (A) Phenotype of two R26-DTR-155T founders. LN cells of Foxp3-GFP-Cre x R26-YFP × R26-DTR-155T mice were stained for DTR. YFP^+^ cells represent Cre expressing and once expressed cells, which were divided into two populations, DTR-expressing cells (low miR-155-5p expression) and DTR-non-expressing cells (high miR-155-5p expression). Figure on the right represents DTR MFI of indicated cells. (B) Comparison of DTR expression level between exFoxp3-gated cells and Treg-gated cells from two founders of R26-DTR-155T and one of R26-DTR. (C) Depletion of DTR-expressing Treg cells. The figure on the left represents the percentage of the remaining YFP^+^CD4^+^ cells in peripheral blood. Each mouse was i.p. injected with 50 μg/Kg body weight of DT on two consecutive days, at day0 and day1 after taking blood. YFP^+^CD4^+^ cells represent Treg cells. The figure on the right shows pooled data of depletion efficiency of Treg cells after the first DT injection or 24 hours after DT treatment. Each symbol represents one mouse and small horizontal lines indicate the mean. Significance was calculated by Student’s *t*-test using GraphPad Prism 5 software. N = 3, ns—not significant; ***P<0.001.

Since Treg cells express the highest level of miR-155-5p activity, we respectively crossed two founders (47 and 84) of R26-DTR-155T mice with Foxp3-GFP-Cre x R26-YFP mice to produce triple transgenic mice (Foxp3-**GFP-**Cre x R26-**YFP** × R26-**DTR**-155T mice). The two founders revealed a distinctive expression pattern of DTR with two populations, DTR- expressing (miR-155-5p^low^) Treg cells and DTR- non-expressing (miR-155-5p^high^) Treg cells (Fig 5A). According to Treg cells expressing two distinct populations of DTR expression level, we considered that DTR- expressing Treg cells might be exFoxp3s [28]. These cells were Foxp3^+^ at one time, but do not express detectable Foxp3 anymore. Thus, we pre-fixed cells with 1% paraformaldehyde to preserve YFP and then stained for Foxp3. Using this method, we were able to distinguish Treg cell subset into exFoxp3 cells and Treg cells characterized by YFP^+^Foxp3^-^ and YFP^+^Foxp3^+^ respectively (Fig 5B). Surprisingly, exFoxp3 cells still maintained high level of miR-155-5p activity even after they lost Foxp3 expression (Fig 5B), which is inconsistent with previous study that continuous Foxp3 expression is indispensable for the maintenance of high amounts of miR-155-5p in Treg cells [10]. These results suggested that miR-155-5p can be induced through other signaling molecules such as T-cell receptor [34]. Treg can be divided into central T reg (cTR) and effector T reg (eTR) cells, based upon the expression of CD62L and CD44, which have very different homeostatic characteristics [35]. In consistent with the results in conventional T cells, we found that miR-155-5p activity was significantly high in effector T reg (eTR) cells, which express CD62L^low^ and CD44^high^ cells (Fig 4B and 4D).

To assess whether the DTR-expressing Treg cells would be eliminated, we injected each mouse with DT twice and monitored the number of remaining Treg cells in peripheral blood. The remaining number of Treg cells from Foxp3-GFP-Cre x R26-YFP mice lacking transgene did not change during the time of injection. Approximately 90% of Treg cells from Foxp3-GFP-Cre x R26-YFP × R26-DTR mice lacking miR-155-5p target were depleted whereas two founders 47 and 84 carrying miR-155-5p target had 55% and 25% of eliminated Treg cells, respectively, which were consistent with the number of DTR- expressing Treg cells (Fig 5C). These results demonstrated that R26-DTR-155T mice provided cell ablation in distinctive miR-155-5p expressing cells.

## Discussion

A new challenge in the field of miRNA biology is to study its function in living systems. miRNA activity is likely to be influenced by multiple factors. Schug et. al. reported that miRNA recruitment to the RISC did not always correlate with the level of miRNA expression [18]. Thus, we speculated that it would be informative to assess miRNA activity by detecting output of expression level of reporter protein in living single cell. To study miR activity *in vivo*, we generated a novel miR-155-5p transgenic mouse line by inserting miR-155 -5p target sequence downstream of a ubiquitous expressing reporter comprising DTR and BFP based on miR-OFF system. To gain both efficiency and sensitivity, we chose BAC transgenic approach to generate our miR-155-5p transgenic mouse line, termed as R26-DTR-155T. A previously validated murine BAC clone RP24-85L1 was used to generate R26-DTR and R26-DTR-155T BAC transgenic strains in this study [36]. Giel-Moloney et al [36] had demonstrated the ubiquitous and uniform expression of transgene under control of this 187 kb BAC containing the R26 locus. Moreover, multiple copies of BAC transgene can be integrated into mouse chromosome after pronuclear microinjection, which can significantly increase the transgene expression level [31]. We found that DTR expression from CMV-Cre x R26-DTR mice indeed recapitulated the expression pattern of R26 knockin strain. However, DTR expression from R26-DTR-155T mice was inversely correlated with miR-155-5p expression in most of the cell types. Moreover, decreased DTR expression can be partial recovered by inhibition of endogenous miR-155-5p activity. Thus, this new miR-155-5p transgenic mouse line could be used to track miR-155-5p activity *in vivo* in specific cell types of interest.

Currently, we do not know a threshold level of miR-155-5p activity that is required for playing a critical role in cell lineage differentiation and immune responses to control physiological and pathological processes. It has been reported that miR-155 promotes autoimmune diseases in experimental autoimmune encephalomyelitis (EAE) model [37–39]. Thus the R26-DTR-155T mice might be useful to address this question by using adoptive transfer of cells with distinct level of miR-155-5p activity into recipient mice.

In addition, using R26-DTR-155T mice as a miR-155-5p activity sensor, we showed that effector cells defined by CD44^high^ or CD62L^low^ cells express higher level of miR-155 -5p than naïve cells. It has now become clear that miR-155-5p levels are increased after stimulation of cells through multiple cellular signaling pathways and the activation of T cells is a tightly regulated process to maximize protective immune responses to pathogens while minimizing damage to self-tissues [40]. The role of miR-155-5p activity after cell activation is unclear, suggesting that these new miR-155-5p transgenic mice may prove to be useful tools based on tissue-specific miR-155-5p activity sensor. OX40 (CD134) is a member of the TNF receptor family and constitutively expressed on activated cells. It is strongly induced in activated CD4 T cells and Treg cells in OX40 Cre mice [41]. Another gene, the orphan nuclear hormone receptor Nur77 (also known as NR4A1), is rapidly induced in response to BCR signaling. Zikherman and colleagues [42] used the Nur77-GFP transgenic reporter mice to track BCR signaling during B cell development, showing that B cell responsiveness to antigen receptor stimulation was found to vary in a manner that was inversely correlated with GFP signal. Thus, both OX40 and Nur77 reporter mice are suitable to study miR-155-5p activity in activated CD4^+^ T cells, Treg cells, and activated B cells respectively, by crossing with our miR-155-5p transgenic mice. The role of miR-155-5p in activated CD8^+^ T cells was studied in the context of antiviral T cell responses *in vivo* [12]. It was shown that effector T cells have higher miR-155-5p expression level than naïve cells and effector memory (T_EM_) cells by maintaining miR-155-5p at a higher level, while central memory (T_CM_) cells downregulate miR-155-5p expression to the same level in naïve cells, suggesting that miR-155-5p might indeed play a functional role in CD8^+^ memory differentiation. After pathogen clearance, it is known that most of the specific T cells undergo apoptosis and some develop into memory cells but we know little about the role of miR-155-5p in memory fate decision. With potential to deplete naïve cells and central memory cells, miR-155-5p transgenic mice might be used to monitor the fate of effector and effector memory CD8^+^ T cells. Further studies would be required for confirming whether DTR expression from the miR-155-5p transgenic mice is only regulated by miR-155-5p, and it can be achieved by crossing the miR-155 deficient mice or miRNA-deficient mice with R26-DTR-155T mice.

One drawback of R26-DTR-155T mice is the relative long half-life of DTR-BFP (S1 Fig), which might not be suitable for monitoring dynamic processes of miR-155-5p. In such cases, DTR-BFP reporter can be substituted with d1GFP variant in the future, which has a much shorter half-life [43].

In conclusion, we propose that the R26-DTR-155T mice might prove to be efficient *in vivo* model for studying distinctive miR-155-5p expressing cell subsets. In addition, we expect that these mice would be useful tools for tracking miR-155-5p activity in activated cells in several different experimental contexts.

## Supporting Information

S1 Fig Validation of DTR turnover by cycloheximide assay. HEK293T cells transfected with pDTR.BFP.155T-N1. 48 hours post transfection, cells were treated with 100 μg/mL of cycloheximide for consecutive 0, 4, 8, 12, 24, and 48 hours. The treated cells were stained for DTR protein expression and analyzed by flow cytometry.
(TIF)

## References

[1] Lagos-Quintana M, Rauhut R, Yalcin A, Meyer J, Lendeckel W, Tuschl T. Identification of Tissue-Specific MicroRNAs from Mouse. Current Biology. 2002;12(9):735–9. 12007417 10.1016/s0960-9822(02)00809-6

[2] Weber MJ. New human and mouse microRNA genes found by homology search. FEBS. 2005;272(1):59–73. 15634332 10.1111/j.1432-1033.2004.04389.x

[3] Faraoni I, Antonetti FR, Cardone J, Bonmassar E. miR-155 gene: a typical multifunctional microRNA. Biochimica et Biophysica Acta. 2009;1792(6):497–505. 10.1016/j.bbadis.2009.02.013 19268705

[4] Elton TS, Selemon H, Elton SM, Parinandi NL. Regulation of the MIR155 host gene in physiological and pathological processes. Gene. 2013;532(1):1–12. 10.1016/j.gene.2012.12.009 23246696

[5] Elton TS, Sansom SE, Martin MM. Cardiovascular Disease, Single Nucleotide Polymorphisms; and the Renin Angiotensin System: Is There a MicroRNA Connection? International Journal of Hypertension. 2010 Oct 16. 10.4061/2010/281692 PMC2949081 20948563

[6] Costinean S, Zanesi N, Pekarsky Y, Tili E, Volinia S, Heerema N, et al Pre-B cell proliferation and lymphoblastic leukemia/high-grade lymphoma in E(mu)-miR155 transgenic mice. Proceedings of the National Academy of Sciences of the United States of America. 2006;103(18):7024–9. 16641092 10.1073/pnas.0602266103 PMC1459012

[7] Moffett HF, Novina CD. A small RNA makes a Bic difference. Genome Biology. 2007;8(7):221 17666120 10.1186/gb-2007-8-7-221 PMC2323214

[8] Rodriguez A, Vigorito E, Clare S, Warren MV, Couttet P, Soond DR, et al Requirement of bic/microRNA-155 for normal immune function. Science. 2007;316(5824):608–11. 17463290 10.1126/science.1139253 PMC2610435

[9] Trotta R, Chen L, Ciarlariello D, Josyula S, Mao C, Costinean S, et al miR-155 regulates IFN-gamma production in natural killer cells. Blood. 2012;119(15):3478–85. 10.1182/blood-2011-12-398099 22378844 PMC3325038

[10] Lu LF, Thai TH, Calado DP, Chaudhry A, Kubo M, Tanaka K, et al Foxp3-dependent microRNA155 confers competitive fitness to regulatory T cells by targeting SOCS1 protein. Immunity. 2009;30(1):80–91. 10.1016/j.immuni.2008.11.010 19144316 PMC2654249

[11] Baumjohann D, Ansel KM. MicroRNA-mediated regulation of T helper cell differentiation and plasticity. Nature Reviews Immunology. 2013;13(9):666–78. 10.1038/nri3494 23907446 PMC3980848

[12] Gracias DT, Stelekati E, Hope JL, Boesteanu AC, Doering TA, Norton J, et al The microRNA miR-155 controls CD8(+) T cell responses by regulating interferon signaling. Nature Immunology. 2013;14(6):593–602. 10.1038/ni.2576 23603793 PMC3664306

[13] Vigorito E, Perks KL, Abreu-Goodger C, Bunting S, Xiang Z, Kohlhaas S, et al microRNA-155 regulates the generation of immunoglobulin class-switched plasma cells. Immunity. 2007;27(6):847–59. 18055230 10.1016/j.immuni.2007.10.009 PMC4135426

[14] Thai TH, Calado DP, Casola S, Ansel KM, Xiao C, Xue Y, et al Regulation of the germinal center response by microRNA-155. Science. 2007;316(5824):604–8. 17463289 10.1126/science.1141229

[15] Tili E, Croce CM, Michaille JJ. miR-155: on the crosstalk between inflammation and cancer. International Reviews of Immunology. 2009;28(5):264–84. 10.1080/08830180903093796 19811312

[16] Kong W, Zhao J-J, He L, Cheng JQ. Strategies for profiling MicroRNA expression. Journal of Cellular Physiology. 2009;218(1):22–5. 10.1002/jcp.21577 18767038

[17] Friedlander MR, Chen W, Adamidi C, Maaskola J, Einspanier R, Knespel S, et al Discovering microRNAs from deep sequencing data using miRDeep. Nature Biotechnology. 2008;26(4):407–15. 10.1038/nbt1394 18392026

[18] Schug J, McKenna L, Walton G, Hand N, Mukherjee S, Essuman K, et al Dynamic recruitment of microRNAs to their mRNA targets in the regenerating liver. BMC Genomics. 2013;14(1):264. 23597149 10.1186/1471-2164-14-264 PMC3639193

[19] Jeker LT, Bluestone JA. MicroRNA regulation of T-cell differentiation and function. Immunological Reviews. 2013;253(1):65–81. 10.1111/imr.12061 23550639 PMC3621017

[20] Mansfield JH, Harfe BD, Nissen R, Obenauer J, Srineel J, Chaudhuri A, et al MicroRNA-responsive 'sensor' transgenes uncover Hox-like and other developmentally regulated patterns of vertebrate microRNA expression. Nature Genetics. 2004;36(10):1079–83. 15361871 10.1038/ng1421

[21] Brown BD, Venneri MA, Zingale A, Sergi Sergi L, Naldini L. Endogenous microRNA regulation suppresses transgene expression in hematopoietic lineages and enables stable gene transfer. Nature Medicine. 2006;12(5):585–91. 16633348 10.1038/nm1398

[22] Lee JY, Kim S, Hwang do W, Jeong JM, Chung JK, Lee MC, et al Development of a dual-luciferase reporter system for in vivo visualization of MicroRNA biogenesis and posttranscriptional regulation. Journal of Nuclear Medicine. 2008;49(2):285–94. 10.2967/jnumed.107.042507 18199619

[23] Wang G, Dong X, Hu J, Tian W, Yuchi J, Wang Y, et al Long-term ex vivo monitoring of in vivo microRNA activity in liver using a secreted luciferase sensor. Science China Life Sciences. 2011;54(5):418–25. 10.1007/s11427-011-4171-0 21574043

[24] Amendola M, Giustacchini A, Gentner B, Naldini L. A double-switch vector system positively regulates transgene expression by endogenous microRNA expression (miR-ON vector). Molecular therapy: Journal of the American Society of Gene Therapy. 2013;21(5):934–46. 10.1038/mt.2013.12 PMC3666624 23439497

[25] Ezzine S, Vassaux G, Pitard B, Barteau B, Malinge JM, Midoux P, et al RILES, a novel method for temporal analysis of the in vivo regulation of miRNA expression. Nucleic Acids Research. 2013;41(20):e192 10.1093/nar/gkt797 24013565 PMC3814383

[26] Srinivas S, Watanabe T, Lin CS, William CM, Tanabe Y, Jessell TM, et al Cre reporter strains produced by targeted insertion of EYFP and ECFP into the ROSA26 locus. BMC Developmental Biology. 2001;1:4 11299042 10.1186/1471-213X-1-4 PMC31338

[27] Murai K, Murakami H, Nagata S. Myeloid-specific transcriptional activation by murine myeloid zinc-finger protein 2. Proceedings of the National Academy of Sciences of the United States of America. 1998;95(7):3461–6. 9520388 10.1073/pnas.95.7.3461 PMC19858

[28] Zhou X, Bailey-Bucktrout SL, Jeker LT, Penaranda C, Martinez-Llordella M, Ashby M, et al Instability of the transcription factor Foxp3 leads to the generation of pathogenic memory T cells in vivo. Nature Immunology. 2009;10(9):1000–7. 10.1038/ni.1774 19633673 PMC2729804

[29] Schwenk F, Baron U, Rajewsky K. A cre-transgenic mouse strain for the ubiquitous deletion of loxP-flanked gene segments including deletion in germ cells. Nucleic Acids Research. 1995;23(24):5080–1. 8559668 10.1093/nar/23.24.5080 PMC307516

[30] Nehlsen K, Schucht R, da Gama-Norton L, Kromer W, Baer A, Cayli A, et al Recombinant protein expression by targeting pre-selected chromosomal loci. BMC Biotechnology. 2009;9:100 10.1186/1472-6750-9-100 20003421 PMC2804664

[31] Alexander GM, Erwin KL, Byers N, Deitch JS, Augelli BJ, Blankenhorn EP, et al Effect of transgene copy number on survival in the G93A SOD1 transgenic mouse model of ALS. Brain research Molecular Brain Research. 2004;130(1–2):7–15. 15519671 10.1016/j.molbrainres.2004.07.002

[32] Kuchen S, Resch W, Yamane A, Kuo N, Li Z, Chakraborty T, et al Regulation of microRNA expression and abundance during lymphopoiesis. Immunity. 2010;32(6):828–39. 10.1016/j.immuni.2010.05.009 20605486 PMC2909788

[33] Seddiki N, Swaminathan S, Phetsouphanh C, Kelleher AD. miR-155 is differentially expressed in Treg subsets, which may explain expression level differences of miR-155 in HIV-1 infected patients. Blood. 2012;119(26):6396–7. 10.1182/blood-2012-02-412874 22745300

[34] Das LM, Torres-Castillo MD, Gill T, Levine AD. TGF-beta conditions intestinal T cells to express increased levels of miR-155, associated with down-regulation of IL-2 and itk mRNA. Mucosal Immunology. 2013;6(1):167–76. 10.1038/mi.2012.60 22785227 PMC3504619

[35] Smigiel KS, Richards E, Srivastava S, Thomas KR, Dudda JC, Klonowski KD, et al CCR7 provides localized access to IL-2 and defines homeostatically distinct regulatory T cell subsets. Journal of Experimental Medicine. 2014;211(1):121–36. 10.1084/jem.20131142 24378538 PMC3892972

[36] Giel-Moloney M, Krause DS, Chen G, Van Etten RA, Leiter AB. Ubiquitous and uniform in vivo fluorescence in ROSA26-EGFP BAC transgenic mice. Genesis. 2007;45(2):83–9. 17269129 10.1002/dvg.20269 PMC2121618

[37] O'Connell RM, Kahn D, Gibson WS, Round JL, Scholz RL, Chaudhuri AA, et al MicroRNA-155 promotes autoimmune inflammation by enhancing inflammatory T cell development. Immunity. 2010;33(4):607–19. 10.1016/j.immuni.2010.09.009 20888269 PMC2966521

[38] Murugaiyan G, Beynon V, Mittal A, Joller N, Weiner HL. Silencing microRNA-155 ameliorates experimental autoimmune encephalomyelitis. Journal of Immunology. 2011;187(5):2213–21. 10.4049/jimmunol.1003952 21788439 PMC3167080

[39] Zhang J, Cheng Y, Cui W, Li M, Li B, Guo L. MicroRNA-155 modulates Th1 and Th17 cell differentiation and is associated with multiple sclerosis and experimental autoimmune encephalomyelitis. Journal of Neuroimmunology. 2014;266(1–2):56–63. 10.1016/j.jneuroim.2013.11.009 24332164

[40] Lind EF, Ohashi PS. Mir-155, a central modulator of T-cell responses. European Journal of Immunology. 2014;44(1):11–5. 24571026 10.1002/eji.201343962

[41] Klinger M, Kim JK, Chmura SA, Barczak A, Erle DJ, Killeen N. Thymic OX40 expression discriminates cells undergoing strong responses to selection ligands. Journal of Immunology. 2009;182(8):4581–9. 10.4049/jimmunol.0900010 19342632 PMC2736618

[42] Zikherman J, Parameswaran R, Weiss A. Endogenous antigen tunes the responsiveness of naive B cells but not T cells. Nature. 2012;489(7414):160–4. 10.1038/nature11311 22902503 PMC3438375

[43] Corish P, Tyler-Smith C. Attenuation of green fluorescent protein half-life in mammalian cells. Protein Engineering. 1999;12(12):1035–40. 10611396 10.1093/protein/12.12.1035

