# Supplementary figures and images for: A Novel Transgenic Mouse Line for Tracing MicroRNA-155-5p Activity In Vivo
Source: PLoS One. 2015 Jun 1;10(6):e0128198. doi: 10.1371/journal.pone.0128198 (PMC4452368; doi:10.1371/journal.pone.0128198)

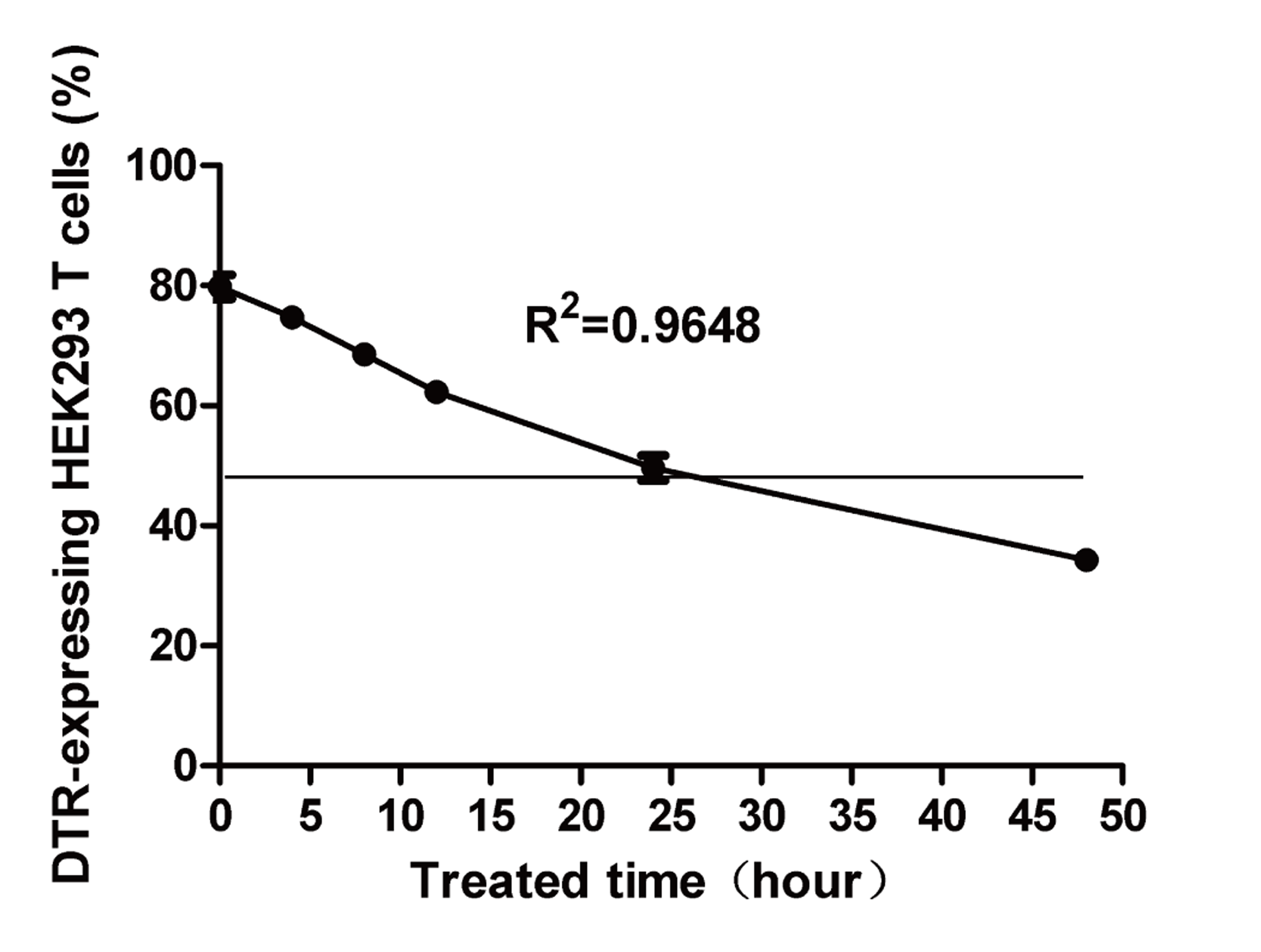

Supplement: S1 Fig — HEK293T cells transfected with pDTR.BFP.155T-N1. 48 hours post transfection, cells were treated with 100 μg/mL of cycloheximide for consecutive 0, 4, 8, 12, 24, and 48 hours. The treated cells were stained for DTR protein expression and analyzed by flow cytometry. (TIF) [file pone.0128198.s001.tif]
